# Growth and dietary effects on gastrointestinal symptoms in children with Hirschsprung’s disease

**DOI:** 10.1007/s00383-026-06657-3

**Published:** 2026-09-26

**Authors:** Lovisa Telborn, Irene Axelsson, Christina Granéli, Pernilla Stenström

**Affiliations:** 1 Department of Pediatric Surgery, Skåne University Hospital, Klinikgatan 15, Lund, S-222 42 Sweden; 2 Department of Clinical Sciences, Pediatrics, Lund University, Lund, Sweden

**Keywords:** Hirschsprung’s disease, Diet, Gastrointestinal symptoms, Growth

## Abstract

**Purpose:**

Growth and diet are major concerns for patients with Hirschsprung’s disease yet are rarely described. This study aimed to investigate self-reported growth and the dietary impact on gastrointestinal symptoms in children with Hirschsprung’s disease.

**Methods:**

This observational cross-sectional study used a validated self-report questionnaire. All children aged 1–18 years old (*n* = 85) with Hirschsprung’s disease treated at a national center were invited to participate.

**Results:**

In total, 71 children (84%) participated (median age 6 years [range 1–17]). Stunting was reported in 6 of 71 children (9%), with no significant difference between those with rectosigmoid and long-segment aganglionosis (4/58 [7%] vs. 2/13 [15%], *p* = 0.328). The majority (55/71; 77%) reported diet-induced gastrointestinal symptoms, again with no significant difference between the groups (43/58 [74%] vs. 12/13 [92%] *p* = 0.227). Of the 90 food items assessed, the most frequently reported to trigger symptoms were high in fermentable oligosaccharides, disaccharides, monosaccharides, and polyols (FODMAPs), free sugar and fat.

**Conclusion:**

Diet-related gastrointestinal complaints are reported frequently in children with Hirschsprung’s disease, regardless of aganglionic extent. The findings highlight the need to assess growth and dietary factors in follow-up care and support further interventional studies on dietary strategies.

**Supplementary Information:**

The online version contains supplementary material available at https://doi.org/10.1007/s00383-026-06657-3.

## Introduction

Hirschsprung’s disease is a congenital intestinal disorder characterized by aganglionosis of the intestinal wall, extending proximally from the anus to varying lengths of bowel [1–5]. Rectosigmoid aganglionosis accounts for 80–85% of cases, long-segment aganglionosis for approximately 10%, and total colonic aganglionosis for fewer than 10% of cases [1, 4, 6–8]. Hirschsprung’s disease requires surgical management, with removal of the affected part of the bowel [1, 5]. However, despite surgery, residual gastrointestinal symptoms, such as constipation, outlet obstruction, fecal incontinence, abdominal pain and flatulence are reported frequently [1–8]. To improve bowel function, the European consensus guidelines for the management of rectosigmoid Hirschsprung’s disease recommend dietary modifications as part of the bowel management program [1]. While short-term growth at 1 year of age is generally within normal limits regardless of aganglionic segment length [9], children with long-segment aganglionosis are at increased risk of long-term faltering growth and nutritional deficiencies [6–7, 10–12]. Accordingly, both the European guidelines for rectosigmoid Hirschsprung’s disease and the clinical consensus statements on total colonic and intestinal aganglionosis recommend regular growth monitoring during follow-up [1, 8]. Although dietary concerns are highly relevant to patients and families [13], few studies have examined the impact of diet on gastrointestinal symptoms or the nutritional needs of children with Hirschsprung’s disease [6–12, 14–15]. Evidence-based dietary management strategies remain lacking. Previous research has shown that a substantial proportion (77%) of children with Hirschsprung’s disease report diet-induced gastrointestinal symptoms and often engage in dietary self-management [14–16]. Families have also expressed a strong need for dietary guidance to manage these symptoms [13–15, 17]. Importantly, the dietary impact on gastrointestinal symptoms in children with Hirschsprung’s disease appears to differ significantly from that in healthy peers. These children with Hirschsprung’s disease not only experience symptoms more frequently but also report different types of diet-related effects [15]. This distinction highlights the need for disease-specific dietary guidelines. To improve clinical counseling and inform future interventional studies on growth and dietary management in Hirschsprung’s disease, it is essential to expand our understanding of growth patterns and the role of specific dietary components on gastrointestinal symptomatology.

## Aim

This study aimed to investigate self-reported growth status and perceptions of whether diet and specific food items trigger gastrointestinal symptoms in children with Hirschsprung’s disease.

## Materials and methods

### Study setting

This was an observational, cross-sectional, patient-reported questionnaire study conducted at a pediatric surgery department serving as a national referral center for Hirschsprung’s disease since 2018, covering a catchment area of approximately 5 million residents. Data collection took place from March 2020 to March 2021.

### Study questionnaire

**Fig. 1:**
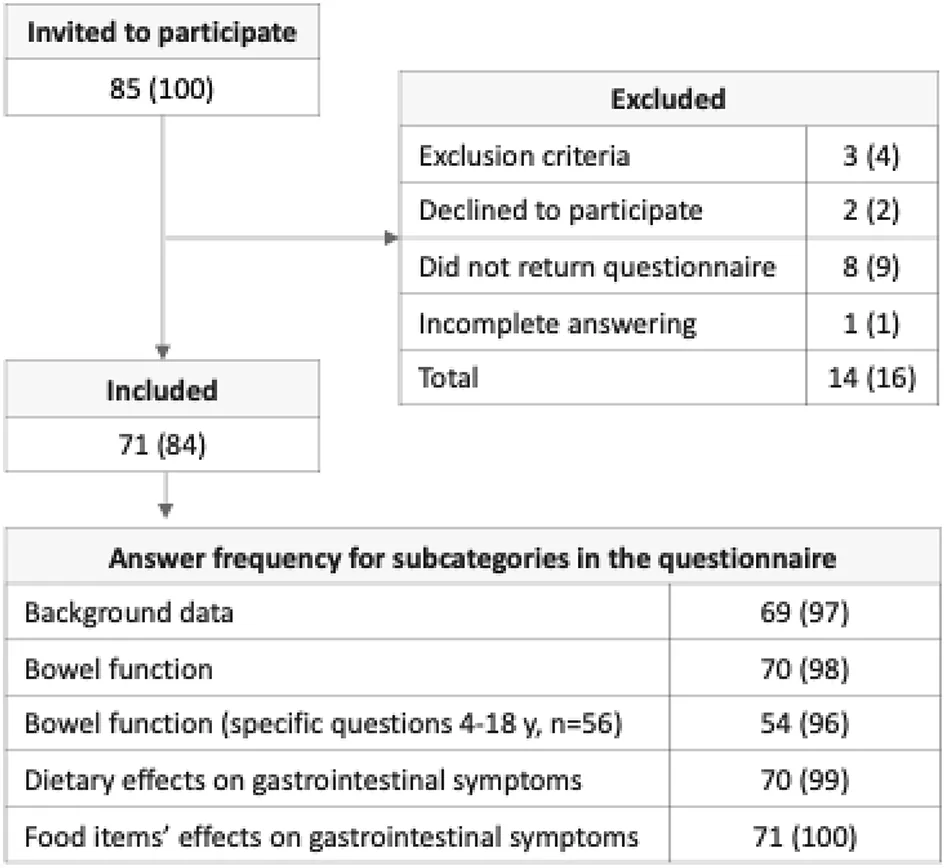
Flowchart of participants: children with Hirschsprung’s disease 1–18 years old included and excluded in the study and answer frequency for each category in the questionnaire. Values are *n* (%) of children

The validated questionnaire included 32 questions in total (as shown in Online Resource 1) [18]. Nine questions addressed growth, comorbidities, medications, and allergies. Growth parameters (current length/height and weight) were self-reported by patients or their parents and evaluated according to World Health Organization (WHO) growth classification standards [19–23]. Gastrointestinal symptoms were assessed using the Rintala Bowel Function Score (BFS) [24–25] with additional questions on constipation, abdominal pain, and bothersome gases [7]. The BFS, commonly used in children with Hirschsprung’s disease, consists of seven questions with a total score ranging from 1 to 20 (20 = best function). A score of 18–20 indicates normal bowel function, while 1–17 reflects impaired or moderately impaired bowel function [24–25]. Dietary effects on gastrointestinal symptoms were assessed using the Diet and Bowel Function questionnaire [18], which includes 13 questions and 90 specified food items. Respondents indicated whether each food item caused any gastrointestinal symptoms and, if so, the type (i.e. any, laxative, constipating, abdominal pain, gas, or other). To explore potential mechanisms, food items were categorized based on their content into six groups: Fermentable Oligosaccharides, Disaccharides, Monosaccharides, And Polyols (FODMAPs); fat; free sugar; stringy or hard to digest textures; histamine-releasing or biogenic amine-containing foods. Response frequencies for each category in the questionnaire are shown in Fig. 1. Parents were instructed to complete the questionnaire by proxy for young children and to complete the questionnaire together with their child for children younger than 15 years. Adolescents aged 15–18 years were encouraged to complete the questionnaire independently.

### Study population

All children aged 1–18 years treated for Hirschsprung’s disease at the department were invited to participate, along with their parents. Study invitations and questionnaires were distributed by postal mail. Children receiving enteral tube feeding or parenteral nutrition were excluded.

### Statistical analysis

A statistician designed the statistical analysis plan. Only questionnaires with at least 90% of questions completed (≥ 29/32) were included. For BFS analysis, only participants aged 4–18 years who had completed all BFS questions were included. Descriptive data are presented as *n* (%). Data on a continuous variable (i.e. age), is reported as median (range) due to non-normal distribution. The nonparametric Mann-Whitney *U* test was used for continuous and discrete (number of food items reported to induce gastrointestinal symptoms) variables and for graded data on an ordinal scale. Binary categorical variables were analyzed using the Chi-square test. A *p*-value < 0.05 was considered statistically significant. Analyses were performed using IBM SPSS Statistics version 29 for MacOS.

Hirschsprung’s disease was classified as rectosigmoid aganglionosis (*n* = 58), long-segment aganglionosis extending beyond the rectosigmoid colon to the descending or transverse colon (*n* = 8), or total colonic aganglionosis, including extension of aganglionosis up to 20 cm into the ileum (*n* = 5). Rectosigmoid aganglionosis served as the reference group, as this is the most common subtype of Hirschsprung’s disease. Long-segment and total colonic aganglionosis were analyzed together, as the phenotypes were few and analyses of the distribution of bowel function outcomes showed no differences between the groups. Univariate subgroup analyses (including BFS, age and sex) were conducted for dietary effects on gastrointestinal symptoms and dietary adjustments. No significant differences were found within subgroups (Online Resource 2). Therefore, subsequent analyses of self-reported growth and dietary effects on gastrointestinal symptoms were stratified only by aganglionic extent (rectosigmoid vs. long-segment aganglionosis). Responses to questions on constipation and outlet obstruction were dichotomized as “No” versus “Yes” (including symptoms without treatment, or treated with diet, medication, enemas, or other methods), soiling (Often/Always), abdominal pain (Often/Always) and bothersome gases (Often/Always).

### Ethical considerations

The study was conducted in accordance with the Declaration of Helsinki and was approved by the Swedish Ethical Review Authority (registration number 2018/720). Participants received age-appropriate oral and written information. Informed consent was obtained from both legal guardians for children under 15 years of age, while adolescents aged 15 years or older provided their own consent.

## Results

### Background information and gastrointestinal symptoms

A total of 71 out of 85 children (84%) met the inclusion criteria (i.e. ≥90% of questions answered and no enteral or parenteral nutrition). A flowchart of participant inclusion and exclusion is shown in Fig. 1. The median age of respondents was 6 years (range 1–17), and 56 of 71 (79%) were boys. Among participants, 58 (82%) had rectosigmoid aganglionosis and 13 (18%) had long-segment aganglionosis, including five (7%) with total colonic aganglionosis. Three participants (4%) had an ostomy. The BFS was completed by 50 children aged 4–18 years. Normal bowel function (BFS 18–20) was reported by 15 of 50 (30%) participants, with no significant difference between children with rectosigmoid and long-segment aganglionosis (14/42 [33%] vs. 1/8 [13%], (*p* = 0.407). A full overview of BFS outcomes is provided in Online Resource 3. The self-reported gastrointestinal symptoms obstruction, abdominal pain and bothersome gases were analyzed for all 71 participants aged 1–18 years. Obstructive symptoms were reported by 26 (37%), abdominal pain by 14 (20%) and bothersome gases by 37 (52%), with no significant differences between aganglionosis subtypes (Online Resource 4).

### Growth and nutritional supplements

Stunting was reported by 6 of 71 children (9%) children, with no significant difference between those with rectosigmoid and long-segment aganglionosis (4/58 [7%] vs. 2/13 [15%], *p* = 0.328). Growth status based on weight and self-reported history of faltering growth also did not differ significantly between groups (Table 1). Children with long-segment aganglionosis more frequently reported current or previous intake of nutritional supplements compared to those with rectosigmoid aganglionosis (8/13 [62%] vs. 12/58 [21%], (*p* = 0.007).

**Table 1:** Growth status according to WHO classification, self-reported growth impairment and need of nutritional supplements reported by children with Hirschsprung’s disease 1–18 years old, using the Diet and Bowel function questionnaire

Growth status and need of nutritional supplements	All children with HSCR *n* = 71	Rectosigmoid aganglionosis *n* = 58	Long-segment aganglionosis *n* = 13	*p*-value
*Growth status according to WHO classifications*				
Stunted^1^	6 (9)	4 (7)	2 (15)	0.328^13^
*Missing*	*9 (13)*	*8 (14)*	*1 (8)*	
Underweight/Wasted^2,3^	2 (3)	2 (3)	0 (0)	0.567^14^
Healthy weight^4,5,6^	47 (66)	37 (64)	10 (77)	
Overweight^7,8,9^	9 (13)	8 (14)	1 (8)	
Obesity^10,11,12^	2 (3)	2 (3)	0 (0)	
*Missing*	*11 (15)*	*9 (16)*	*2 (15)*	
*Self-reported growth impairment*				
Impaired weight gain	17 (24)	13 (22)	4 (31)	0.496^13^
*Missing*	*0 (0)*	*0 (0)*	*0 (0)*	
Impaired growth (length/height)	14 (20)	11 (19)	3 (23)	0.698^15^
*Missing*	*2 (3)*	*1 (2)*	*1 (8)*	
*Need of nutritional supplements*				
Never	51 (72)	46 (79)	5 (39)	0.007^14^
Not currently, but have in the past	13 (18)	7 (12)	6 (46)	
Occasionally need	5 (7)	3 (5)	2 (15)	
Regularly need	2 (3)	2 (3)	0 (0)	
*Missing*	*0 (0)*	*0 (0)*	*0 (0)*	

*WHO*, World Health Organization, *HSCR* Hirschsprung’s disease

^1^Length/Height for age − 2SD or below

^2^Children 1 year old: Weight for length − 2SD or below

^3^Children 2–18 years old: BMI for age − 2SD or below

^4^Children 1 year old: Weight for length − 2SD – <2 + SD

^5^Children 2–5 years old: BMI for age − 2SD – <+2SD

^6^Children 5–18 years old: BMI for age − 2SD – <+1SD

^7^Children 1 year old: Weight for length +2SD – <+3SD

^8^Children 2–5 years old: BMI for age +2SD – <+3SD

^9^Children 5–18 years old: BMI for age +1SD – <+2SD

^10^Children 1 year old: Weight for length +3SD or above

^11^Children 2–5 years old: BMI for age +3SD or above

^12^Children 5–18 years old: BMI for age +2SD or above

^13^Fischer’s exact test

^14^Mann-Whitney *U* Tests, two-tailed

^15^Chi-square test

### Food items inducing gastrointestinal symptoms

The majority of children (55/71; 77%) reported that diet induced gastrointestinal symptoms, with no significant difference between aganglionosis subtypes (43/58 [74%] vs. 12/13 [92%] *p* = 0.227). In total, 58 of 71 children (82%) identified at least one food item that triggered symptoms. The median number of symptom-inducing food items reported was seven (range 0–66). Children with long-segment aganglionosis reported a significantly higher number of symptom-inducing foods compared to children with rectosigmoid aganglionosis (median 18 [0–66] vs. 6 [0–36] (*p* = 0.038). Specific food items reported to induce gastrointestinal symptoms varied between children reporting symptoms of soiling, obstruction, gases, constipation, respectively, abdominal pain (Table 2). Banana, corn and beans were reported frequently as inducing gastrointestinal symptoms across all symptom types. Unique triggers for soiling included pear, apple, cauliflower and popcorn; for gases strawberry and orange; and for abdominal pain, lactose-free milk, carrots, peppers, butter, and Swedish crispbread. The three most frequently reported symptom-inducing foods overall were milk, banana and corn (Table 3). The top 10 food items associated with each gastrointestinal symptom type are listed in Table 3.

**Table 2:** Food items reported to induce any (unspecified) gastrointestinal effect, reported by more than 20% children with Hirschsprung’s disease (*n* = 71) 1–18 years old with gastrointestinal symptoms (soiling, obstruction, gases, constipation, abdominal pain) using the Diet and Bowel function questionnaire

Children with soiling *n* = 25		Children with obstruction *n* = 24		Children with gases *n* = 37		Children with constipation *n* = 23		Children with abdominal pain *n* = 14	
Banana	10 (40)	Beans	11 (46)	Milk	19 (51)	Beans	11 (48)	Milk	7 (50)
Corn	10 (40)	Banana	10 (42)	Banana	15 (41)	Banana	9 (39)	Beans	5 (36)
Beans	9 (36)	Corn	10 (42)	Corn	14 (38)	Corn	9 (39)	Cream	5 (36)
Cream	9 (36)	Fruit peel	6 (25)	Cream	14 (38)	White bread	7 (30)	Yoghurt	5 (36)
Milk	8 (32)	Milk	6 (25)	Yoghurt	14 (38)	Cabbage	6 (26)	Bread: grains/seeds	5 (36)
Onion	8 (32)	Cabbage	5 (21)	Ice cream	13 (35)	Onion	5 (22)	Pastry	5 (36)
Ice cream	8 (32)	Onion	5 (21)	White bread	12 (32)			Chocolate	5 (36)
Yoghurt	8 (32)	Potato	5 (21)	Candy	12 (32)			Candy	5 (36)
White bread	8 (32)	Yoghurt	5 (21)	Clementine	11 (30)			Corn	4 (29)
Pastry	7 (28)			Beans	10 (27)			Ice cream	4 (29)
Chips	7 (28)			Onion	10 (27)			Lactose-free milk	4 (29)
Bread: grains/seeds	6 (24)			Fruit peel	10 (27)			White bread	4 (29)
Candy	6 (24)			Bread: grains/seeds	10 (27)			Banana	3 (21)
Deep fried food	6 (24)			Strawberry	9 (24)			Clementine	3 (21)
Pear	5 (20)			Pastry	9 (24)			Carrot	3 (21)
Apple	5 (20)			Chips	9 (24)			Pepper	3 (21)
Cauliflower	5 (20)			Chocolate	9 (24)			Potato	3 (21)
Fruit peel	5 (20)			Deep fried food	9 (24)			Fruit peel	3 (21)
Chocolate	5 (20)			Orange	8 (22)			Butter	3 (21)
Popcorn	5 (20)							Swedish cracker	3 (21)
								Chips	3 (21)
*Median (range) number of food items reported per child*									
7 (0–36)		6 (0–24)		10 (0–60)		7 (0–36)		8 (0–22)	

Values are *n* (%) of children

**Table 3:** Ranking of the 10 most frequently reported food items to induce any (unspecified) gastrointestinal effect, laxative effect, constipating effect, rising pain and/or rising gases respectively, reported by children with Hirschsprung’s disease (*n* = 71) 1–18 years old using the Diet and Bowel function questionnaire

Ranking	Any effect		Laxative effect		Constipating effect		Rising pain		Rising gases	
1	Milk	26 (37)	Cream	15 (21)	Banana	20 (28)	Milk	9 (13)	Beans	18 (25)
2	Banana	24 (34)	Ice cream	13 (18)	White bread	13 (18)	Corn	8 (11)	Milk	14 (20)
3	Corn	24 (34)	Milk	12 (17)	Rice	8 (11)	Cream	6 (9)	Onion	11 (16)
4	Cream	23 (32)	Candy	12 (17)	Corn	7 (10)	Beans	5 (7)	Cabbage	11 (16)
5	Beans	21 (30)	Yoghurt	11 (16)	Potato	7 (10)	Yoghurt	5 (7)	Cream	9 (13)
6	Yoghurt	21 (30)	Kiwi	10 (14)	Pasta	7 (10)	Ice cream	5 (7)	Yoghurt	8 (11)
7	Ice cream	19 (27)	Pastry	9 (13)	Flour	6 (9)	Candy	5 (7)	Ice cream	8 (11)
8	White bread	19 (27)	Plum	8 (11)	Apple	5 (7)	Pastry	5 (7)	Cauliflower	8 (11)
9	Candy	18 (25)	Chocolate	7 (10)	Chips	4 (6)	Chips	4 (6)	Chips	6 (9)
10	Pastry	17 (24)	Clementine	7 (10)	Fruit peel	4 (6)	Chocolate	4 (6)	Deep fried food	6 (9)

Values are *n* (%) of children

### Potential underlying mechanisms of symptom-inducing foods

The most frequently reported food category to induce gastrointestinal symptoms was FODMAPs. Among the 10 most frequently reported symptom-inducing foods, nine were high in FODMAPs. The second and third most reported categories were foods high in free sugar and high in fat, respectively (Table 4). Across all 90 food items assessed, FODMAPs remained the most frequently reported category reported to induce gastrointestinal symptoms (Online Resource 5).

**Table 4:** Potential underlying mechanism of inducing gastrointestinal symptoms as reported by children with Hirschsprung’s disease 1–18 years old using the Diet and Bowel function questionnaire

Potential underlying mechanism	Number of food items in the questionnaire (In total *n* = 90)	Frequency of children (*n* = 71) reporting at least one food item	Median number of food items reported per child	Food items included in the 10 most frequently reported food items to induce gastrointestinal symptoms (number and specific food item)	
FODMAPs	40	46 (65%)	4 (range 0–32)	9	Milk, banana, corn, cream, beans, yoghurt, ice cream, white bread, pastry
High content of fat	6	33 (46%)	1 (range 0–6)	3	Cream, ice cream and pastry
High content of free sugar	5	30 (42%)	0 (range 0–5)	3	Ice cream, candy and pastry
Stringy foods or foods hard to digest	33	37 (52%)	2 (range 0–24)	2	Corn and beans
Histamine releasing	10	32 (45%)	1 (range 0–9)	1	Milk
Biogenic amines	8	37 (52%)	1 (range 0–7)	1	Banana

*FODMAPs* Fermentable Oligosaccharides, Disaccharides, Monosaccharides, and Polyols

### Dietary adjustments to improve gastrointestinal complaints

Overall, 49 of 71 children (69%) reported making dietary adjustments to manage gastrointestinal symptoms. The most common reasons were to obtain looser stools (23/49; 47%), reduce flatulence (20/49; 41%) and to obtain a constipating effect (13/49; 27%). A total of 27 children (38%) reported actively *choosing* specific foods or dietary habits to improve gastrointestinal symptoms. The most frequently chosen foods were fruits (i.e. kiwi, pear, banana, prune, grapes, blueberries, apple, plum, and apricot) reported by 9 of 27 children (33%). Probiotics were used by six (22%) of the children, and a lactose-free diet was followed by eight (30%). Avoidance of specific foods or dietary habits was reported by 44 of 71 children (62%). Of these, 42 children specified the foods avoided. The most avoided foods were lactose-containing foods (16/42; 38%), fruit peel, stringy or hard-to-digest foods (10/42; 24%), and free sugar- or fiber-rich foods (8/42; 19%).

### Free-text comments on the influence of diet on gastrointestinal symptoms

Free-text comments were provided by 46/71 (65%) respondents and revealed three main themes. Portion size was most frequently reported (24/46, 52%), with respondents describing limiting rather than excluding foods associated with symptoms. Selective eating (11/46, 24%) made it difficult to evaluate foods that were rarely consumed, or not part of the child’s diet, particularly fruits and vegetables. In addition, 9/46 (20%) respondents reported difficulty in identifying consistent associations between specific foods and gastrointestinal symptoms.

## Discussion

This self-reported study found that 9% of children with Hirschsprung’s disease reported growth status classified as stunted growth and 28% required nutritional supplementation. Supplement use was significantly more common among children with long-segment aganglionosis compared to children with rectosigmoid extension. Overall, 77% of participants reported that diet induced gastrointestinal symptoms, with no differences based on aganglionic length. Foods high in FODMAPs, free sugar and fat were most frequently reported as gastrointestinal symptom triggers.

The prevalence of stunting was higher in children with long-segment aganglionosis (15%) than in those with rectosigmoid extension (7%), and both rates exceeded the 2.2% prevalence reported in the general pediatric population, based on the WHO definition (≥ 2 standard deviations below the mean) [26]. Previous studies have reported faltering growth in 23–55% of children with total colonic aganglionosis [6–7, 10–12], and an association between faltering growth and aganglionic extent has been suggested [10]. The lower prevalence observed in our study may be due to the inclusion of only children with total colonic aganglionosis extending no more than 20 cm into the small bowel, who were grouped with those with milder long-segment aganglionosis. Additionally, 61% of children with long-segment aganglionosis in our study reported using nutritional supplements, compared to 47% in a previous Nordic study [7]. At our center, all children receiving supplements are followed regularly by both a pediatric dietitian and a pediatrician, which may have contributed to better growth outcomes. Interpretation of the growth findings is limited by the use of self-reported data. Objective measurements are needed to assess the prevalence of faltering growth accurately. The etiology of faltering growth in Hirschsprung’s disease is likely to be multifactorial, including repeated surgical interventions and admissions for enterocolitis, familial short stature, endocrine or genetic conditions, and dietary restrictions to manage gastrointestinal symptoms may all play a role. Further studies are needed to investigate the relationship between dietary intake, nutritional status, and growth in this population.

A key finding was that 77% of children reported diet-induced gastrointestinal symptoms, consistent with previous studies in children and adults with Hirschsprung’s disease (64–77%) [16, 27–29]. A novel observation was that symptom-triggering foods varied according to the type of gastrointestinal complaint, supporting individualized dietary counseling. Dietary modifications may be particularly relevant in relation to soiling. Families may modify the diet to promote bowel motility and facilitate stool passage; however, the same foods may increase stool frequency or loosen stool consistency and thereby contribute to soiling. Conversely, foods perceived to aggravate soiling may already have been limited or excluded from the child’s diet. This makes the relationship between dietary intake, bowel function, and soiling complex and potentially bidirectional, and should be considered when interpreting the reported associations between specific foods and gastrointestinal symptoms. Dietary modifications in response to gastrointestinal symptoms were reported for 69% of the children, consistent with the previously reported rate of 61–72% [16, 29]. In our cohort, 38% reported selecting specific foods, whereas 62% avoided certain foods. However, whether these dietary modifications were associated with symptom improvement was not assessed. Future studies could explore associations between individual foods, or combination of foods, and gastrointestinal symptoms. The rationale for dietary modifications, including whether they were medically advised or self-initiated, was not assessed. However, European and Latin American studies have suggested that dietary modifications among patients with Hirschsprung’s disease were prominently self-directed [16, 29]. This calls for awareness in clinical counseling. Foods high in FODMAPs, free sugar, and fat were most frequently reported as symptom triggers, although overlap between food categories complicates interpretation. The observed associations are biologically plausible. FODMAPs increase luminal water content and undergo bacterial fermentation, resulting in loose stools, gas production, and abdominal distension [30]. Free sugar and fat may also exacerbate symptoms through stimulation of the gastrocolonic response and postprandial colonic motility [31], which may be particularly problematic in children with persistent bowel dysfunction after Hirschsprung’s disease surgery [1–7]. A small exploratory study reported improved fecal soiling in 9 of 10 children with Hirschsprung’s disease and fecal incontinence following a low-FODMAP diet [32]. Although low-FODMAP diets may benefit children with irritable bowel syndrome, routine use is not currently recommended because of their restrictive nature and limited evidence base [30]. When implemented in children, the diet should follow a structured approach consisting of a 2–6-week FODMAP-restriction phase followed by systematic reintroduction to identify individual trigger foods and achieve the least restrictive long-term diet [30]. Dietary assessment and follow-up by a dietitian are recommended to ensure nutritional adequacy [30]. Whether low-FODMAP or other dietary approaches are beneficial in Hirschsprung’s disease should be evaluated in well-designed interventional studies. The present findings provide a basis for such research.

As this study was designed to evaluate patient-reported experiences regarding diet and gastrointestinal symptoms, the findings should be interpreted in the context of other factors known to influence gastrointestinal symptoms in children with Hirschsprung’s disease. Several disease-specific mechanisms contribute to postoperative gastrointestinal symptoms. Surgical treatment alters anorectal anatomy and physiology, which may impair continence and bowel function [2, 5]. Reduced anal sensation, absence of internal anal sphincter relaxation, and a preserved aganglionic cuff may contribute to fecal incontinence and/or outlet obstruction [2, 5]. In addition, many patients exhibit accelerated colonic transit, lack of the rectal brake, an exaggerated colonic response, and a hyperreactive colon, which may increase postprandial urgency, stool frequency, abdominal distension, and fecal soiling [33–34]. Therefore, the results do not imply that dietary factors are the sole or primary drivers of gastrointestinal symptoms.

The strengths of this study include the use of a validated, disease-specific questionnaire [18], detailed data on food-related gastrointestinal symptoms, and a high response rate from a national referral center. These factors ensured broad representation across age, sex, symptom severity, and aganglionic extent. However, potential response bias cannot be excluded, as families with food-related concerns may have been more likely to participate. Several limitations should be considered. The main limitation was the small sample size in subgroup analyses, which may have limited the ability to detect differences between aganglionosis types. Due to the small sample sizes, patients with long-segment aganglionosis and total colonic aganglionosis were combined into one group, as no functional outcomes were observed between these subgroups. However, we acknowledge that grouping these phenotypes may be questioned, as they may have distinct clinical courses. The findings should therefore be interpreted with caution. Larger, multicenter studies are needed to confirm whether dietary effects on gastrointestinal symptoms vary by disease severity. The information provided with the questionnaire instructed parents and school-aged children up to 15 years of age to complete the questionnaire together. For the youngest children, parents were instructed to respond by proxy, while the oldest adolescents were encouraged to complete the questionnaire independently. As only one questionnaire was distributed per household, parents and children did not provide separate responses, precluding comparisons between parent-proxy and child-reported responses. Furthermore, whether these instructions were followed was not recorded. Consequently, the extent to which the responses reflect the children’s own experiences cannot be determined. These aspects should be considered potential limitations of the study. Furthermore, the cross-sectional design precludes conclusions regarding causality. Notably, some respondents reported that they were unable to evaluate the effects of specific foods because these foods were not consumed by the child. This highlights an inherent challenge in assessing and reporting food-related effects, as dietary habits may already have been modified in response to previous gastrointestinal symptoms, or dietary restrictions might be due to avoidant/restrictive food intake disorder. Consequently, the reported effects of specific foods may not fully reflect their potential impact on symptoms. Postoperative complications, such as bowel obstruction or anastomotic strictures, were not assessed in the present study. Such complications may influence both gastrointestinal symptoms and dietary intake, for example by leading to dietary modifications or avoidance of certain foods. The lack of information on previous postoperative complications, and the frequency of these in the cohort, could therefore be considered a limitation of the study. Gastrointestinal symptoms were assessed prospectively using the Rintala Bowel Function Score, which is used widely in pediatric surgery and has been shown to capture key domains of bowel function in patients with Hirschsprung’s disease. The use of the Rome IV criteria [35] would have facilitated broader interpretation and comparison of our findings, as these criteria are well established and used widely among pediatricians. The lack of assessment according to the Rome IV criteria should therefore be considered a limitation, particularly when comparing our findings with studies from the field of pediatric gastroenterology.

Children with Hirschsprung’s disease present with a wide range of gastrointestinal symptoms. For effective clinical counseling, it is essential to recognize that not all symptoms can be managed through diet alone. For example, pain and gases may result from outlet obstruction requiring surgical intervention, and incontinence may be the result of a low anastomosis, necessitating bowel management strategies beyond dietary changes [2, 5]. A multidisciplinary approach, including individualized dietary counseling by a pediatric dietitian experienced in Hirschsprung’s disease, is essential. To develop evidence-based dietary recommendations, interventional studies are needed. The present findings offer a strong foundation for future clinical trials.

## Conclusion

Diet-related gastrointestinal symptoms are reported frequently in children with Hirschsprung’s disease, regardless of aganglionic extent. Foods with a high content of FODMAPs, free sugar and fat appear to be particularly problematic. The findings support the inclusion of multidisciplinary discussions on diet and routine assessment of growth and dietary habits in follow-up care. Interventional studies on dietary treatment strategiesare warranted.

## Supplementary Information

Below is the link to the electronic supplementary material.

Supplementary Material 1

Supplementary Material 2

Supplementary Material 3

Supplementary Material 4

Supplementary Material 5

## Data Availability

All data supporting the findings of this study are available on request.

## Declarations

### Standards of reporting

Reporting guidelines for observational studies (STROBE) were followed.
