# Supplementary material for: Growth and dietary effects on gastrointestinal symptoms in children with Hirschsprung’s disease
Source: Pediatr Surg Int. 2026 Sep 26;42(1):425. doi: 10.1007/s00383-026-06657-3 (PMC13615995; doi:10.1007/s00383-026-06657-3)
Supplement: Supplementary file 1 [file 383_2026_6657_MOESM1_ESM.pdf]

Online Resource 4. Gastrointestinal symptoms in children aged 1-18 years old with Hirschsprung's disease using the Diet and Bowel Function questionnaire. Values are n (%) of children.

	All children n=71	HSCR Short-segment n=58	HSCR Long-segment n=13	p-value
**Obstruction**				
Never	43 (61)	34 (59)	9 (69)	
Obstruction without treatment	6 (9)	5 (9)	1 (8)	p=0.118
Manageable with diet	2 (3)	2 (3)	0 (0)	
Manageable with medicine	13 (18)	12 (21)	1 (8)	
Not manageable with either diet or medicine	5 (7)	5 (9)	0 (0)	
Missing	2 (3)	0 (0)	2 (15)	
**Abdominal pain**				
Never	22 (31)	19 (33)	3 (21)	
Sometimes (at most once a week)	35 (49)	27 (47)	8 (62)	
Often	14 (20)	12 (21)	2 (15)	p=0.802
Always	0 (0)	0 (0)	0 (0)	
Missing	0 (0)	0 (0)	0 (0)	
**Bothersome gases**				
Never	6 (9)	6 (10)	0 (0)	
Sometimes (at most once a week)	28 (39)	21 (36)	7 (54)	
Often	31 (44)	26 (45)	5 (39)	p=0.923
Always	6 (9)	5 (9)	1 (8)	
Missing	0 (0)	0 (0)	0 (0)	

HSCR, Hirschsprung's disease
