# Supplementary material for: Growth and dietary effects on gastrointestinal symptoms in children with Hirschsprung’s disease
Source: Pediatr Surg Int. 2026 Sep 26;42(1):425. doi: 10.1007/s00383-026-06657-3 (PMC13615995; doi:10.1007/s00383-026-06657-3)
Supplement: Supplementary file 2 [file 383_2026_6657_MOESM2_ESM.pdf]

Online Resource 1. The Diet and Bowel function questionnaire. Questions and answering options.

Background data	Gastrointestinal symptoms and Bowel Function Score	Dietary habits and gastrointestinal symptoms	Specific food items and gastrointestinal symptoms
**1. Age**<br>(Years)<br>**2. Gender**<br>(Girl/Boy)<br>**3. Height**<br>(cm)<br>**4. Weight**<br>(kg)<br>**5a. Do you have any disease, allergy or any gastrointestinal congenital malformation?**<br>(Yes/No)<br>**5b. If yes, What allergy/disease?**<br>(Open answer)<br>**5c. What is the gastrointestinal congenital malformation?**<br>(Open answer)<br>**6a. Do you take any treatment for bowel symptoms?**<br>(Yes/No)<br>**6b. If yes, What type of treatment?**<br>(Adjusted diet/Medicine (e.g., for diarrhea or constipation, antibiotics, analgesic), What type of medicine?/Enemas/Other reason)<br>**6c. If yes, for what reason?**<br>(Constipation/Diarrhea/Stomach pain/Other reason)<br>**7. Have you ever had impaired growth?** (Weight Yes/No, Height Yes/No)<br>**8. Have you ever had the need for nutritional supplements**<br>**(e.g., oral nutritional supplements)?**<br>(No, never, Not currently, but I have in the past, Yes, occasionally need, Yes, need regularly)<br>**9a. Do you have any special diet? (e.g., vegetarian, vegan)**<br>(Yes/no)<br>**9b. If yes, What type of special diet?**<br>(Open answer)	**10a. How often do you/your child poop?** (Every other day to twice a day/More often than twice a day/Less often than every other day)<br>**10b. How many times a week do you/your child poop?**<br>(Open answer)<br>**11. Do you/does your child have hard poop and troubles to push it out?** (No/ Yes, but it passes without doing anything/ Yes, then I need to eat prunes, pears, kiwis or other food to make the poop softer/ Yes, I need to eat medicine that makes the poop softer/ Yes, I need to use medicine in my butt to be able to push the poop out)<br>**12. Do you/does your child ever experience difficulties in school or in their spare time, for example doing what you/your child wants, or seeing friends, due to problems with stools coming in underwear or the risk of it happening?** (No social problems/ Sometimes/ Often/ Daily)<br>**13. Do you/does your child feel the urge to poop and verbalizes when you/he or she will poop?**<br>(Always/Most of the time/Not often/seldom/Never)<br>**14. Are you/your child able to hold back defecation?**<br>(Yes, always or almost always/ Problems less than 1 time per week/ Weekly/ No, my child has no or little voluntary control)<br>**15. Do you/does your child ever soil (staining in underwear)?** (Never or very seldom/Less than 1 time per week no change of underwear required/Often, change of underwear required many times per week/Daily, requires protective aids)<br>**16. Do you/does your child ever have accidents where a lot of poop comes out in the underwear?**<br>(Never/Less than 1 time per week/Weekly, often require protective aids/Daily, protective aids required day and night)<br>**17. Do you experience abdominal pain?** (Never/ Sometimes [at most once a week]/ Frequently/Always)<br>**18. Do you suffer from bothersome gases?** (Never/ Sometimes [at most once a week]/Frequently/Always)<br>**19. Do you experience difficulties in passing stools despite soft poop?** (No, never/Yes, without treatment/Yes, manageable with diet/Yes, manageable with medicine/Yes, not manageable with either diet and/or medicine)	**20. Would you agree that your diet affects your stomach?**<br>**(e.g. constipation, diarrhea or bloatedness)**<br>(No, never/Yes, sometimes/Yes, often/Yes, always/Not currently but I have in the past/Please explain how)<br>**21. Would you agree that how you eat affects your stomach?**<br>(No, never/Yes, sometimes/Yes, often/Yes, always/Not currently but I have in the past/Please explain how)<br>**22a. Do you adjust your diet for your stomach's sake?**<br>(No, never/Yes, sometimes/Yes, often/Yes, always/Not currently but I have in the past/Please explain how)<br>**22b. If yes: Why?**<br>(Laxative effect/Constipating effect/Less gases/Other. If so, what?)<br>**23. Do you choose specific types of food to help your stomach?**<br>(Yes/No)<br>**24. Do you avoid specific types of food to help your stomach?**<br>(Yes/No)<br>**25. Is there anyone else in your family who adjusts their diet to help their stomach?**<br>(Yes/No)<br>**26. Does your diet limit you (in school, when you are with friends or in general)?**<br>(I've never thought about it, so I don't find it relevant/No, never/Yes, sometimes/Yes, often/Yes, always/Not currently but I have in the past/Please explain how)<br>**27. Do you think about how your diet affects your stomach?**<br>(No, never/Yes, sometimes/Yes, often/Yes, always/Not currently but I have in the past/Please explain how)<br>**28. To parents: Do you think about your child's diet and how it affects his/her stomach?**<br>(No, never/Yes, sometimes/Yes, often/Yes, always/Not currently but I have in the past/Please explain how)<br>**29. Does your diet affect you emotionally?**<br>(No, never/Yes, sometimes/Yes, often/Yes, always/Not currently but I have in the past/Please explain how)<br>**30. To parents: Does your child's diet affect you emotionally?**<br>(No, never/Yes, sometimes/Yes, often/Yes, always/Not currently but I have in the past/Please explain how)<br>**31a. Would you be interested in finding out more information about how your diet affects your stomach?**<br>(Yes/No)<br>**31b. If yes: Where or who would you turn to to find out more information?**<br>(Open answer)	**32a. Does the food item affect your stomach?**<br>(For every food item: Yes/No/I don't know)<br>**32b. If yes: In which way?**<br>(For every food item: Laxative effect/Constipating effect/Gives gases/Gives pain/Other. If so, what?)<br><br>**Food items listed:**<br>**Fruits**: pineapple, orange, apricot, banana, clementine, strawberry, kiwi, cherry, lingonberry, melon, nectarine, peach, plum, pear, dried fruit, grapes, apple<br>**Vegetables**: avocado, cauliflower, broccoli, beans, cabbage, lentils, onion, corn, carrot, pepper, parsley, potato, rhubarb, celery, fruit peel, asparagus, mushroom, tomato, peas<br>**Dairy**: cream, ice cream, lactose-free milk, milk, cheese, butter, yoghurt<br>**Bread, flour, rice**: bread with grains/seeds, Swedish cracker, cornmeal, pasta, rice, flour, white bread<br>**Meat, fish, egg**: fish, pork, beef, sausage, chicken, salami, shellfish, egg<br>**Sweets and snacks**: pastry, chips, chocolate, candy, popcorn, rice cakes, pretzel sticks<br>**Beverages**: soda (with sugar), soda (free from sugar), water, carbonated drink, wine/beer, formula<br>**Cooking effects**: deep fried food, spicy food, fried food, soup<br>**Spices and seeds**: curry, cayenne, chili/tabasco, chamomilla, sesame, sunflower seeds, poppy seeds<br>**Nuts etc**: cashew, hazelnut, peanut, chestnut, almond, brazil nut, walnut, soy
