# Supplementary material for: Growth and dietary effects on gastrointestinal symptoms in children with Hirschsprung’s disease
Source: Pediatr Surg Int. 2026 Sep 26;42(1):425. doi: 10.1007/s00383-026-06657-3 (PMC13615995; doi:10.1007/s00383-026-06657-3)
Supplement: Supplementary file 3 [file 383_2026_6657_MOESM3_ESM.pdf]

**Online Resource 5.** Ranking of the 90 food items included in the Diet and Bowel function questionnaire and their potential underlying mechanism to induce gastrointestinal symptoms, reported by children with Hirschsprung's disease (n=71) 1-18 years old.

| Rank | Food item | Potential mechanism | n (%) | Rank | Food item | Potential mechanism | n (%) |
|---|---|---|---|---|---|---|---|
| 1 | Milk | FODMAPs, HR | 26 (37) | 46 | Melon | FODMAPs | 6 (9) |
| 2 | Banana | FODMAPs, BA | 24 (34) | 47 | Asparagus | FODMAPs,STR | 6 (9) |
| 3 | Corn | FODMAPs,STR | 24 (34) | 48 | Rice cakes | | 6 (9) |
| 4 | Cream | FODMAPs | 23 (32) | 49 | Water | | 6 (9) |
| 5 | Beans | FODMAPs,STR | 21 (30) | 50 | Formula | FODMAPs | 6 (9) |
| 6 | Yoghurt | FODMAPs | 21 (30) | 51 | Pineapple | STR | 5 (7) |
| 7 | Ice cream | FODMAPs,SU,F | 19 (27) | 52 | Broccoli | STR | 5 (7) |
| 8 | White bread | FODMAPs | 19 (27) | 53 | Beef | STR | 5 (7) |
| 9 | Candy | SU | 18 (25) | 54 | Sausage | FODMAPs,F | 5 (7) |
| 10 | Pastries | FODMAPs,SU,F | 17 (24) | 55 | Pretzel sticks | | 5 (7) |
| 11 | Chips | (FODMAPs),F | 17 (24) | 56 | Diet soda | | 5 (7) |
| 12 | Fruit peel | STR | 15 (21) | 57 | Fried food | F | 5 (7) |
| 13 | Chocolate | FODMAPs,SU,HR, BA | 15 (21) | 58 | Soup | | 5 (7) |
| 14 | Onion | FODMAPs,STR | 15 (21) | 59 | Chili/tabasco | | 5 (7) |
| 15 | Clementine | STR | 14 (20) | 60 | Cashew | FODMAPs,STR | 5 (7) |
| 16 | Bread: grains/seeds | FODMAPs,STR | 14 (20) | 61 | Fish | HR,BA | 4 (6) |
| 17 | Cabbage | FODMAPs,STR | 13 (18) | 62 | Nectarine | FODMAPs | 3 (4) |
| 18 | Deep fried food | F | 13 (18) | 63 | Mushroom | FODMAPs,STR | 3 (4) |
| 19 | Orange | STR,HR,BA | 12 (17) | 64 | Cornmeal | FODMAPs | 3 (4) |
| 20 | Strawberry | HR | 11 (16) | 65 | Chicken | STR | 3 (4) |
| 21 | Kiwi | | 11 (16) | 66 | Salami | FODMAPs,BA | 3 (4) |
| 22 | Plum | FODMAPs | 11 (16) | 67 | Curry | | 3 (4) |
| 23 | Pear | FODMAPs | 11 (16) | 68 | Cayenne | | 3 (4) |
| 24 | Grapes | STR | 11 (16) | 69 | Peanut | STR | 3 (4) |
| 25 | Apple | FODMAPs | 11 (16) | 70 | Apricot | FODMAPs | 2 (3) |
| 26 | Flour | FODMAPs | 11 (16) | 71 | Lingonberry | | 2 (3) |
| 27 | Popcorn | STR | 11 (16) | 72 | Pork | HR | 2 (3) |
| 28 | Cauliflower | FODMAPs,STR | 10 (14) | 73 | Shellfish | HR | 2 (3) |
| 29 | Soda | SU | 10 (14) | 74 | Soy | FODMAPs | 2 (3) |
| 30 | Potato | FODMAPs | 9 (13) | 75 | Cherry | FODMAPs | 1 (1) |
| 31 | Peas | FODMAPs,STR | 9 (13) | 76 | Peach | FODMAPs | 1 (1) |
| 32 | Lactose-free milk | | 9 (13) | 77 | Avocado | FODMAPs | 1 (1) |
| 33 | Butter | F | 9 (13) | 78 | Rhubarb | BA | 1 (1) |
| 34 | Swedish cracker | FODMAPs | 9 (13) | 79 | Celery | FODMAPs,STR | 1 (1) |
| 35 | Pasta | FODMAPs | 9 (13) | 80 | Chamomile | | 1 (1) |
| 36 | Rice | | 9 (13) | 81 | Sesame | STR | 1 (1) |
| 37 | Dried fruit | FODMAPs,STR,S | 8 (11) | 82 | Sunflower seeds | STR | 1 (1) |
| 38 | Lentils | FODMAPs,STR | 8 (11) | 83 | Hazelnut | STR | 1 (1) |
| 39 | Carrot | STR | 8 (11) | 84 | Parsley | | 0 (0) |
| 40 | Tomato | STR,HR,BA | 8 (11) | 85 | Wine/beer | HR,BA | 0 (0) |
| 41 | Cheese | FODMAPs,BA | 8 (11) | 86 | Poppy seeds | STR | 0 (0) |
| 42 | Egg | HR | 8(11) | 87 | Chestnut | STR | 0 (0) |
| 43 | Spicy food | | 8 (11) | 88 | Almond | STR | 0 (0) |
| 44 | Pepper | STR | 7 (10) | 89 | Brazil nut | STR | 0 (0) |
| 45 | Carbonated drink | | 7 (10) | 90 | Walnut | STR | 0 (0) |

Values are n (%) of children.
FODMAPs, Fermentable Oligosaccharides, Disacccharides, Monosaccharides, And Polyols. STR, Stringy food or food hard to digest. SU, food with a high content of sugar. F, food with a high content of fat. HR, Histamine Releasing food. BA, Biogenic Amines.
