# Supplementary material for: Growth and dietary effects on gastrointestinal symptoms in children with Hirschsprung’s disease
Source: Pediatr Surg Int. 2026 Sep 26;42(1):425. doi: 10.1007/s00383-026-06657-3 (PMC13615995; doi:10.1007/s00383-026-06657-3)
Supplement: Supplementary file 4 [file 383_2026_6657_MOESM4_ESM.pdf]

Online Resource 2. Dietary effects on gastrointestinal symptoms and dietary adjustments reported by children aged 1-18 years with Hirschsprung's disease with different Bowel Function Score, age and sex using the Diet and Bowel function questionnaire.

	HSCR BFS 1-17 n=35	HSCR BFS 18-20 n=15	p-value	HSCR 1-3 y n=15	HSCR 4-10 y n=40	HSCR 11-18 y n=16	p-value	HSCR girls n=15	HSCR boys n=56	p-value
**Diet affects gastrointestinal symptoms**										
No	8 (23)	3 (20)	0.911[1]	4 (27)	9 (23)	3 (19)	0.741[3]	3 (20)	13 (23)	0.652[1]
Sometimes	14 (40)	7 (47)		5 (33)	18 (45)	6 (38)		5 (33)	24 (43)	
Often	7 (20)	3 (20)		3 (20)	7 (18)	3 (19)		5 (33)	8 (14)	
Always	6 (17)	2 (13)		3 (20)	6 (15)	4 (25)		2 (13)	11 (20)	
Missing	0 (0)	0 (0)		0 (0)	0 (0)	0 (0)		0 (0)	0 (0)	
**Adjusts diet to improve gastrointestinal symptoms**										
No adjustments	8 (23)	7 (47)	0.403[1]	4 (27)	13 (33)	5 (31)	0.848[3]	4 (27)	18 (32)	0.907[1]
Sometimes	13 (37)	3 (20)		5 (33)	13 (33)	5 (31)		5 (33)	18 (32)	
Often	8 (23)	1 (7)		2 (13)	7 (18)	2 (13)		4 (27)	7 (13)	
Always	6 (17)	4 (27)		4 (27)	7 (18)	4 (25)		2 (13)	13 (23)	
Missing	0 (0)	0 (0)		0 (0)	0 (0)	0 (0)		0 (0)	0 (0)	
**Chooses food to improve gastrointestinal complaints**										
Yes	14 (40)	5 (33)	0.656[2]	6 (40)	17 (43)	4 (25)	0.468[2]	7 (47)	20 (36)	0.438[2]
No	21 (60)	10 (67)		9 (60)	23 (57)	12 (75)		8 (53)	36 (64)	
Missing	0 (0)	0 (0)		0 (0)	0 (0)	0 (0)		0 (0)	0 (0)	
**Avoids food to improve gastrointestinal complaints**										
Yes	25 (71)	6 (40)	**0.036**[2]	9 (60)	28 (70)	7 (44)	0.185[2]	7 (47)	37 (66)	0.169[2]
No	10 (29)	9 (60)		6 (40)	12 (30)	9 (56)		8 (53)	19 (34)	
Missing	0 (0)	0 (0)		0 (0)	0 (0)	0 (0)		0 (0)	0 (0)	

[1]Mann-Whitney U-Tests
[2]Chi-square Test
[3]Kruskal Wallis ANOVA
HSCR = Hirschsprung's disease
BFS = Rintala Bowel Function Score
