# Supplementary material for: Growth and dietary effects on gastrointestinal symptoms in children with Hirschsprung’s disease
Source: Pediatr Surg Int. 2026 Sep 26;42(1):425. doi: 10.1007/s00383-026-06657-3 (PMC13615995; doi:10.1007/s00383-026-06657-3)
Supplement: Supplementary file 5 [file 383_2026_6657_MOESM5_ESM.pdf]

Online Resource 3. Bowel function in children aged 4-18 years old with Hirschsprung's disease according to the Rintala Bowel Function Score (BFS). Numbers are (percentage) or as median (range).

	**Score**	**HSCR All children n=50**	**HSCR Short-segment n=42**	**HSCR Long-segment n=8**	**p-value**
**Ability to hold back defecation**					
Always	3	27 (54)	23 (55)	4 (50)	p=0.972
Problems <1/week	2	14 (28)	10 (24)	4 (50)	
Problems >1/week	1	4 (8)	4 (10)	0 (0)	
Never	0	5 (10)	5 (12)	0 (0)	
**Feels the urge to defecate**					
Always	3	27 (54)	24 (57)	3 (38)	p=0.260
Often	2	10 (20)	7 (17)	3 (38)	
Sometimes	1	11 (22)	10 (24)	1 (13)	
Never	0	2 (4)	1 (2)	1 (13)	
**Frequency of defecation**					
Every other day – twice a day	2	29 (58)	25 (60)	4 (50)	p=0.127
More often	1	21 (42)	17 (40)	4 (50)	
Less often	1	0 (0.0)	0 (0)	0 (0)	
**Soiling**					
Never	3	12 (24)	11 (26)	1 (13)	p=0.196
Sometimes (<1/week)	2	15 (30)	14 (33)	1 (13)	
Often	1	17 (34)	12 (29)	5 (63)	
Always	0	6 (12)	5 (12)	1 (13)	
**Accidents**					
Never	3	33 (66)	29 (69)	4 (50)	p=0.406
Sometimes (<1/week)	2	12 (24)	8 (19)	4 (50)	
Often (>1 week)	1	2 (4)	2 (5)	0 (0)	
Daily	0	3 (6)	3 (7)	0 (0)	
**Constipation**					
No constipation	3	33 (66)	27 (64)	6 (75)	p=0.197
Manageable with diet	2	2 (4)	2 (5)	0 (0)	
Manageable with medicine	1	12 (24)	10 (24)	2 (25)	
Not manageable with either diet or medicine	0	3 (6)	3 (7)	0 (0)	
**Social problems**					
No social problems	3	28 (56)	24 (57)	4 (50)	p=0.087
Sometimes	2	13 (26)	11 (26)	2 (25)	
Problems causing restrictions in social life	1	8 (16)	6 (14)	2 (25)	
Severe social and/or psychological problems	0	1 (2)	1 (2)	0 (0)	
**Total Bowel Function Score**					
BFS	1-20	15 (4-20)	16 (4-20)	15 (9-20)	p=0.631

HSCR, Hirschsprung's disease
[1]Mann-Whitney U-Tests, two-tailed.
